# Transcriptional Activity of Predominant *Streptococcus* Species at Multiple Oral Sites Associate With Periodontal Status

**DOI:** 10.3389/fcimb.2021.752664

**Published:** 2021-09-21

**Authors:** Daniel Belstrøm, Florentin Constancias, Merete Markvart, Martin Sikora, Christiane Elisabeth Sørensen, Michael Givskov

**Affiliations:** ^1^Section for Clinical Oral Microbiology, Department of Odontology, Faculty of Health and Medical Sciences, University of Copenhagen, Copenhagen, Denmark; ^2^Laboratory of Food Biotechnology, Department of Health Sciences and Technology, ETH Zürich, Zürich, Switzerland; ^3^Singapore Centre for Environmental Life Sciences Engineering (SCELSE) Nanyang Technological University, Singapore, Singapore; ^4^Lundbeck Foundation GeoGenetics Centre, Globe Institute, University of Copenhagen, Copenhagen, Denmark; ^5^Costerton Biofilm Center, Department of Immunology and Microbiology, Faculty of Health and Medical Sciences, University of Copenhagen, Copenhagen, Denmark

**Keywords:** periodontitis, *Streptococcus*, metagenomics, metatranscriptomics, oral cavity

## Abstract

**Background:**

*Streptococcus* species are predominant members of the oral microbiota in both health and diseased conditions. The purpose of the present study was to explore if different ecological characteristics, such as oxygen availability and presence of periodontitis, associates with transcriptional activity of predominant members of genus *Streptococcus.* We tested the hypothesis that genetically closely related *Streptococcus* species express different transcriptional activities in samples collected from environments with critically different ecological conditions determined by site and inflammatory status.

**Methods:**

Metagenomic and metatranscriptomic data was retrieved from 66 oral samples, subgingival plaque (n=22), tongue scrapings (n=22) and stimulated saliva (n=22) collected from patients with periodontitis (n=11) and orally healthy individuals (n=11). Species-specific transcriptional activity was computed as Log2(RNA/DNA), and transcriptional activity of predominant *Streptococcus* species was compared between multiple samples collected from different sites in the same individual, and between individuals with different oral health status.

**Results:**

The predominant *Streptococcus* species were identified with a site-specific colonization pattern of the tongue and the subgingival plaque. A total of 11, 4 and 2 pathways expressed by *S. parasanguinis, S. infantis* and *S. salivarius*, respectively, were recorded with significantly higher transcriptional activity in saliva than in tongue biofilm in healthy individuals. In addition, 18 pathways, including pathways involved in synthesis of peptidoglycan, amino acid biosynthesis, glycolysis and purine nucleotide biosynthesis expressed by *S. parasanguinis* and 3 pathways expressed by *S. salivarius* were identified with significantly less transcriptional activity in patients with periodontitis.

**Conclusion:**

Data from the present study significantly demonstrates the association of site-specific ecological conditions and presence of periodontitis with transcriptional activity of the predominant *Streptococcus* species of the oral microbiota. In particular, pathways expressed by *S. parasanguinis* being involved in peptidoglycan, amino acid biosynthesis, glycolysis, and purine nucleotide biosynthesis were identified to be significantly associated with oral site and/or inflammation status.

## Introduction

The oral microbiota is comprised by more than 700 different species, which makes it the second most diverse found in the human body (Dewhirst et al., 2010). The composition of the permanent oral microbiota is shaped by thousands of years of co-evolution together with the host (Fellows Yates et al., 2021), which is why the oral microbiota through symbiotic interactions with human natural defense systems is essential for maintenance of oral health (Marsh and Zaura, 2017).

While the oral microbiota is highly individualized and diverse, genus *Streptococcus* is the predominant genus of the oral microbiota across populations and during both healthy and diseased conditions (Chattopadhyay et al., 2019). A likely explanation to these findings is the versatility of *Streptococcus* species, including being facultative anaerobic, which means that they thrive in areas with plenty or lack of oxygen (Abranches et al., 2018). In addition, *Streptococcus* species express specific surface characteristics, which enables them to colonize both teeth and the oral mucosa, thereby protecting them from transfer to the gastrointestinal channel during swallowing (Jenkinson, 1994). Finally, *Streptococcus* species are proficient in carbohydrate metabolism, which is most often the prime nutrient offered by the oral cavity (Takahashi, 2015).

Carbohydrates from food intake and salivary glycoproteins are the main nutrients of *Streptococcus* species, which in addition to providing energy can be stored as intracellular reservoirs (Busuioc et al., 2009). Furthermore, some *Streptococcus* species can deposit carbohydrates extracellularly as part of the oral biofilm matrix, which is why *Streptococcus* species are critically involved in biofilm pathogenicity (Koo et al., 2013). While carbohydrate metabolism of some *Streptococcus* species is limited to a small number of different carbohydrates, other *Streptococcus* species, such as *Streptococcus mutans*, are capable of degrading almost all types of carbohydrates (Moye et al., 2014). Indeed, carbohydrate metabolism performed by *Streptococcus* species is a double-edged sword. On the more negative side, these bacteria degrade carbohydrates anaerobically *via* fermentation processes that locally lead to acid production and a decrease in biofilm fluid pH, which links *Streptococcus* species with dental caries. On the positive side, the acid production resulting from streptococcal carbohydrate fermentation may function as a regulatory mechanism of the biofilm in situations characterized by a transient increase in local pH, for example during inflammatory conditions such as periodontitis (Nyvad and Takahashi, 2020).

In the oral cavity, *Streptococcus* species live closely together in polymicrobial biofilms, shaped by synergistic and antagonistic interactions with other members of the biofilm community (Lamont et al., 2018). Therefore, the interaction of *Streptococcus* species and their phenotypic profile in situations with different ecological conditions should ideally be studied using *in-vivo* clinical samples (Belstrøm, 2020). Indeed, *Streptococcus* species are closely related genetically, which is why it is often not possible to distinguish them at species level, using 16S rDNA methods (Lal et al., 2011). Thus, most information on metabolic characteristics such as carbohydrate metabolism of *Streptococcus* species comes from *in-vitro* culture microbiology, based on analysis of gene expression by *Streptococcus* species grown in either mono- or polymicrobial biofilms (Abranches et al., 2018). Therefore, much is to be learned on how different *Streptococcus* species adapt to changes in ecological conditions, such as presence of chronic periodontal inflammation, in complex biofilms *in-vivo*.

The oral cavity is unique compared to other body sites, since it is composed of different locations, which despite being anatomically close, are determined by critically different ecological conditions (Kilian et al., 2016). For example, the tongue surface is characterized by a slow epithelial turn-over and deep anaerobic crypts, which favors growth of a mature biofilm (Asakawa et al., 2018). On the other hand, in individuals who perform regular oral hygiene, subgingival dental plaque is a more immature biofilm, formed in predominantly aerobic conditions (Berger et al., 2018). Therefore, the tongue and the subgingival biofilm constitute microbial communities shaped by critically different ecological conditions. Saliva is sterile when entering the oral cavity (Schrøder et al., 2017). Accordingly, saliva is a conglomerate of bacteria shed from different oral surfaces, with subgingival dental and tongue biofilm as some of the prime donor sites (Segata et al., 2012). As compared to tongue and subgingival biofilms, the salivary microbiota is primarily planktonic (Belstrøm, 2020). Indeed, *Streptococcus* species are highly abundant in both tongue and subgingival dental biofilms as well as in saliva (Segata et al., 2012), which is why concomitant characterization of *Streptococcus* species in these samples offer the possibility to study how *Streptococcus* species adapt to different ecological conditions.

The purpose the present study was to determine if different ecological characteristics such as oxygen availability and conditions of chronic periodontal inflammation associate with differential transcriptional activities of predominant members of genus *Streptococcus.* We applied paired metagenomics and metatranscriptomics to characterize and compare transcriptional activity of the predominant *Streptococcus* species identified in clinical samples, i.e. tongue biofilm, subgingival dental biofilm and saliva collected from orally healthy individuals and patients with periodontitis. We tested the hypothesis that transcriptional activities of predominant *Streptococcus* species associate with periodontal status.

## Methods

### Study Population and Sample Collection

The present study is part of a series of studies based on characterization of the oral microbiota in samples collected from the same cohort. Thus, the study population and sample collection has been described in detail (Belstrøm et al., 2021). In brief, a total of 66 microbial samples were collected from 11 patients with chronic periodontitis, and 11 orally healthy controls at the Department of Odontology, University of Copenhagen in January 2018 The study was approved by the regional ethical committee (H-16016368) and reported to the local data authorization of University of Copenhagen (SUND-2018-8).

The periodontitis group (M/F: 5/6), out of which four were current smokers, had a mean age of 64 yrs. (55-77 yrs.), The control group (M/F: 4/7) had a mean age of 60 yrs. (50-71 yrs.), and included three daily smokers. Exclusion criteria in both groups: age< 50 yrs., systemic diseases, use of any kind of medication including usage of antibiotics within the last three months.

Microbial samples, subgingival dental plaque (n=22), tongue scrapings (n=22) and stimulated saliva (n=22), were collected between 8:00 AM and 11:00 AM, according to standardized protocols as previously described (Belstrøm et al., 2021). Subgingival plaque and tongue coatings were suspended in 2 mL Sodium Chloride (9mg/mL). Immediately, after collection each sample was divided in two aliquots of 1 mL each, one each for metagenomics and metatranscriptomics analysis. RNAlater (Life Technologies, Denmark) was added to the aliquot allocated for metatranscriptomics and all aliquots were immediately stored at -80°C until further processing.

### Metagenomic and Metatranscriptomic Library Preparation and Sequencing

Raw data were downloaded from NCBI SRA PRJNA678453. The laboratory procedures used for DNA and RNA extraction and sequencing have been described in detail (Belstrøm et al., 2021).

### Read Preprocessing, Taxonomic, and Functional Profiling

Raw sequencing reads pre-processing was performed as described previously (Belstrøm et al., 2021). Briefly, non-biological sequences, low quality RNA reads identified as ribosomal RNA as well as DNA and RNA reads mapping to the human genomes were discarded from the dataset.

Taxonomic composition of metagenomic reads was determined using MetaPhlan (Beghini et al., 2021) (version 3.0.1, 25 Jun 2020), –min_ab 0.000001 using the latest available database i.e., mpa_v30_CHOCOPhlAn_201901). MetaPhlan relies on read mapping against a built-in collection of clade-specific marker genes database, which allows an unambiguous estimation of species relative abundance across samples with a low species resolution false positives rate as compared to read classifiers.

Sample-specific taxonomic profiles generated using MetaPhlAn on metagenomes were used to filter the HUMAnN (Franzosa et al., 2018) built-in pangenomes database to the organism present in the sample. Metagenomic and metatranscriptomic reads were then mapped using Bowtie2 to sample-specific pangenomes. Metagenomic and metatranscriptomic reads failing to align to the pangenome databases were then blasted against UniRef90 using DIAMOND (Buchfink et al., 2015). Hits were counted per gene family and normalized for length and alignment quality. Gene family abundances were then combined into structured pathways using MetaCyc (Caspi et al., 2014) and sum-normalized to relative abundances. Functional gene family tables were regrouped to KEGG’s orthologs and Enzyme (Enzyme commission number, i.e., EC numbers) with the provided humann2 UniRef90_to_KO and UniRef90_to_EC numbers mapping files, respectively (Franzosa et al., 2018).

### Quantification of Species-Specific Transcriptional Activity

We determined species transcriptional activity as described in (Schirmer et al., 2018). Briefly, the pathway copies per million (cpm) proportions is computed for each species in the metagenomes (i.e., DNA samples) and metatranscriptomes (i.e., RNA samples) within all the subjects. Only pathways detected in both metagenomes and metatranscriptomes were considered. Differences in overall species-level transcriptional activity between sites and health status were tested using two-sided nonparametric Kruskal-Wallis tests and the generated p.values were FDR-corrected to adjust for multiple comparisons. Significant differences between groups were considered for p.values lower or equal to 0.05.

## Results

### Site-Specific Distribution of Predominant *Streptococcus* Species in Oral Health

**Figure 1:**
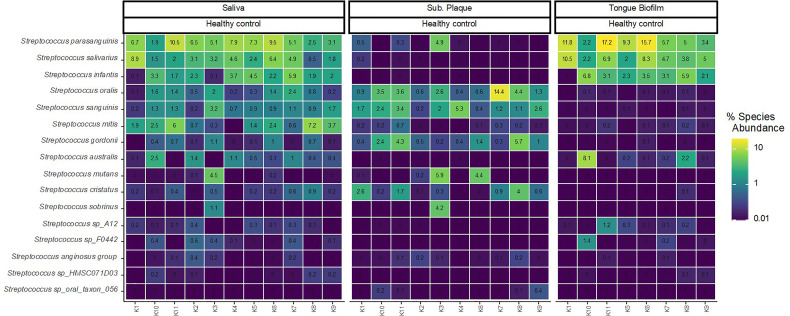
Site-specific distribution of predominant *Streptococcus* species. Relative abundance of the predominant *Streptococcus* species in 29 samples (saliva n = 11, subgingival plaque n = 10, tongue biofilm n = 8) in healthy conditions. Color code denotes the percentage read abundance, where light colors indicates increasing abundance and dark colors decreasing abundance.

**Figure 1** displays relative abundance (DNA) of the 16 predominant *Streptococcus* species across all samples. As can be seen, the proportion of taxa of predominant *Streptococcus* species was significantly influenced by the oral site. Accordingly, *Streptococcus parasanguinis, Streptococcus salivarius and Streptococcus infantis* were the most abundant species identified in tongue biofilm, but almost completely absent in subgingival plaque. On the other hand, *Streptococcus oralis*, *Streptococcus sanguinis* and *Streptococcus gordonii* were all highly abundant in subgingival plaque, and only sporadically present in tongue biofilm. All *Streptococcus* species identified with high abundance in either tongue biofilm or subgingival plaque were concomitantly abundant in saliva samples collected from the same individual.

### Oral Site Associates With Overall Activity of Predominant *Streptococcus* Species in Oral Health

**Figure 2:**
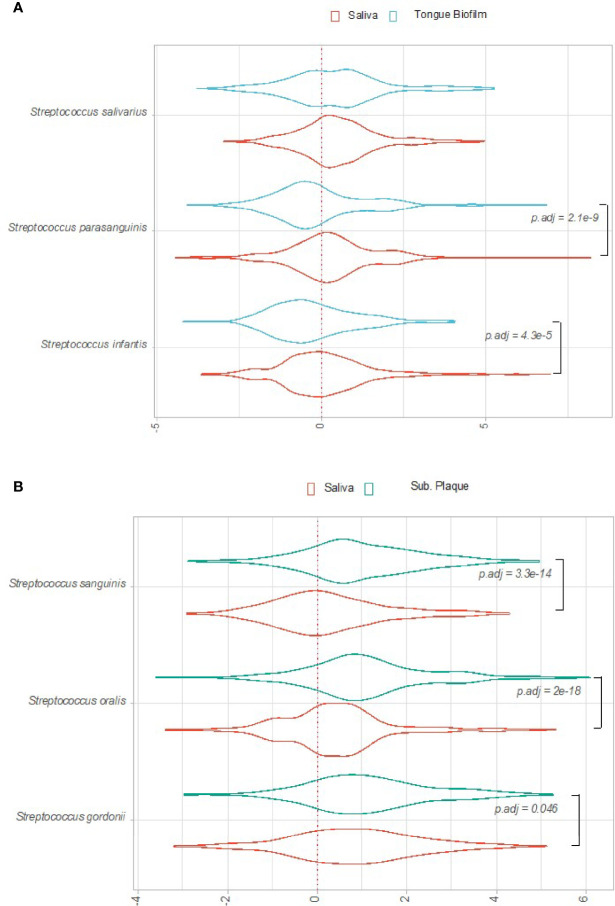
Overall transcriptional activity of predominant *Streptococcus* species in oral health. Transcriptional activity of predominant *Streptococcus* species, as evaluated by log2(RNA/DNA) with each dot representing a specific pathway. **(A)** Saliva versus tongue biofilm. **(B)** Saliva versus subgingival plaque. Color coding: red: saliva, blue: tongue biofilm, green: subgingival plaque.

Bacterial activity, as evaluated by log2(RNA/DNA) of each pathway expressed by the selected predominant *Streptococcus* species, was compared between sites in orally healthy conditions. **Figure 2A** shows the distribution of log2(RNA/DNA) pathway level expression of *S. parasanguinis, S. salivarius and S. infantis* in tongue biofilm versus saliva samples. As can be seen, *S. parasanguinis* and *S. infantis* were significantly more transcriptionally active in saliva than in corresponding tongue biofilm. On the other hand, *S. sanguinis*, *S. oralis* and *S. gordonii* appeared significantly less active in saliva than in subgingival plaque samples collected from the same individual (**Figure 2B**).

### Species-Specific Transcriptional Activity of Specific Pathways Differentiates Between Oral Sites in Health

**Figure 3:**
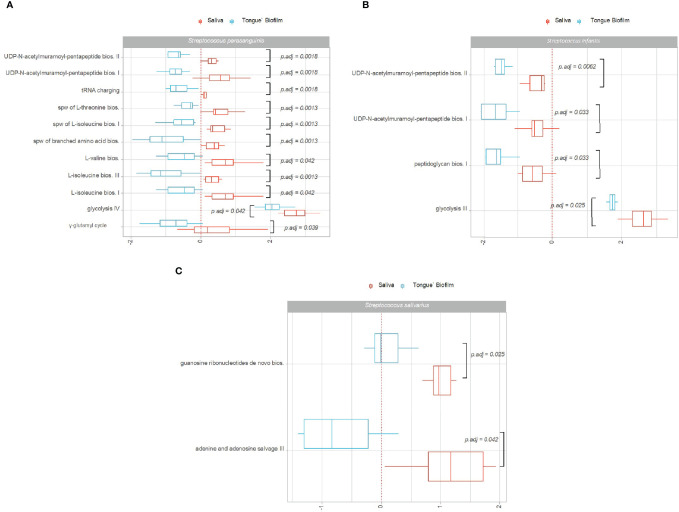
Pathway expression of predominant *Streptococcus* species in saliva versus tongue biofilm. Pathways identified with significantly different log2(RNA/DNA) in saliva versus tongue biofilm in oral health with each dot representing a specific sample. **(A)** *Streptococcus parasanguinis*, **(B)** *Streptococcus infantis*, **(C)** *Streptococcus salivarius*. Boxplots display first and third quartiles, median. Whiskers display 1.5 * IQR. Color coding: red: saliva, blue: tongue biofilm.

**Figures 3A–C** show pathways of *S. parasanguinis, S. infantis* and *S. salivarius*, which were identified with significantly different Log2(RNA/DNA) expression in saliva versus tongue biofilm in healthy individuals. A total of 11, 4 and 2 pathways expressed by *S. parasanguinis, S. infantis* and *S. salivarius*, respectively, were recorded with significantly higher activity in saliva than in tongue biofilm. On the contrary, no pathways were identified with significantly higher expression by *S. parasanguinis, S. infantis* and *S. salivarius* found in tongue biofilm.

**Figure 4:**
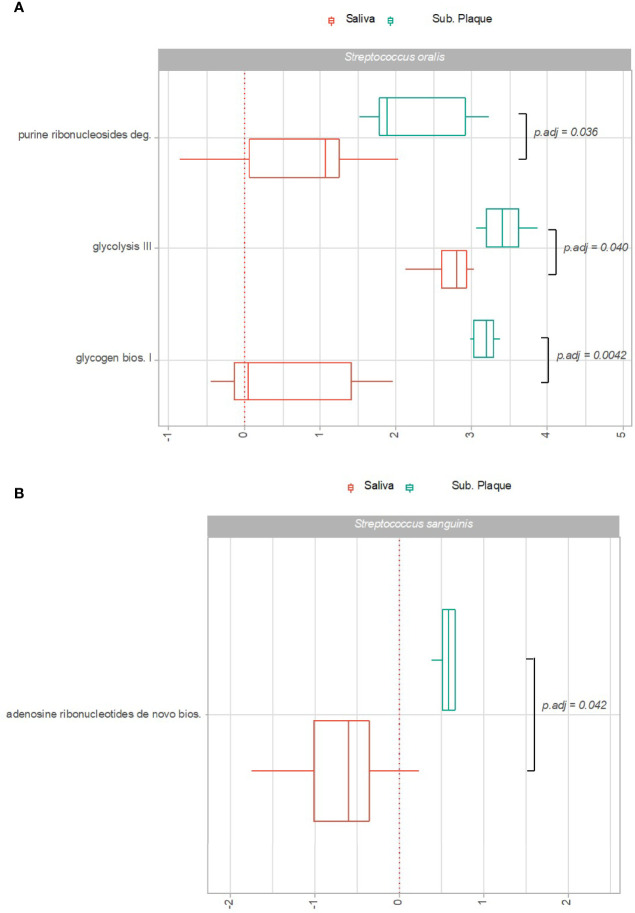
Pathway expression of predominant *Streptococcus* species in saliva versus subgingival plaque. Pathways identified with significantly different log2(RNA/DNA) in saliva versus subgingival plaque in oral health with each dot representing a specific sample. **(A)** *Streptococcus oralis*, **(B)** *Streptococcus sanguinis*. Boxplots display first and third quartiles, median. Whiskers display 1.5 * IQR. Color coding: red: saliva, green: subgingival plaque.

Significant transcriptional activity of pathways expressed by *S. sanguinis* and *S. oralis* in subgingival plaque versus saliva is visualized as Log2(RNA/DNA) in **Figures 4A, B**. As can be seen, 3 pathways expressed by *S. oralis* and one pathway expressed by *S. sanguinis* were observed with significantly higher transcriptional activity in subgingival plaque as compared to saliva in healthy individuals. On the other hand, no pathways were recorded with significantly higher transcriptional activity in saliva. Finally, no pathways were expressed significantly differently by *S. gordonii* in subgingival plaque versus saliva.

### Periodontal Status Associates With Overall Bacterial Activity of Predominant *Streptococcus* Species

**Figure 5:**
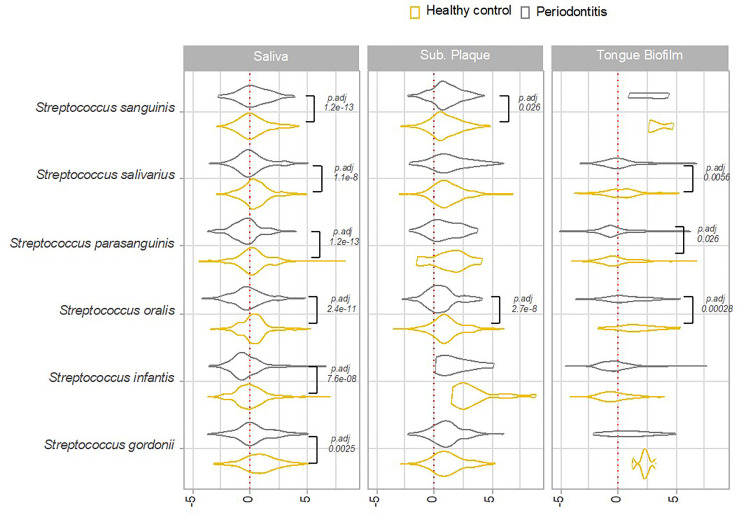
Overall transcriptional activity of *Streptococcus* species in oral health versus periodontitis. Transcriptional activity of predominant *Streptococcus* species in periodontitis versus oral health, as evaluated by log2(RNA/DNA), with each dot representing a specific pathway. Color coding: yellow: healthy control, grey: periodontitis.

In general, higher transcriptional activity of *Streptococcus* species was observed in samples from orally healthy individuals than in patients with periodontitis. Accordingly, a significantly higher transcriptional activity of *S. salivarius*, *S. parasanguinis*, *S. oralis*, *S. infantis* and *S. gordonii* was noted in saliva from healthy controls as compared to patients with periodontitis. Likewise, significantly higher transcriptional activities of *S. salivarius*, *S. parasanguinis*, and *S. oralis* was seen in tongue biofilm from orally healthy individuals versus patients with periodontitis. Finally, a significantly higher activity of *S. oralis* was observed in subgingival plaque from healthy persons (**Figure 5**).

### Species-Specific Transcriptional Activity of Specific Pathways Differentiates Periodontitis From Oral Health

**Figure 6:**
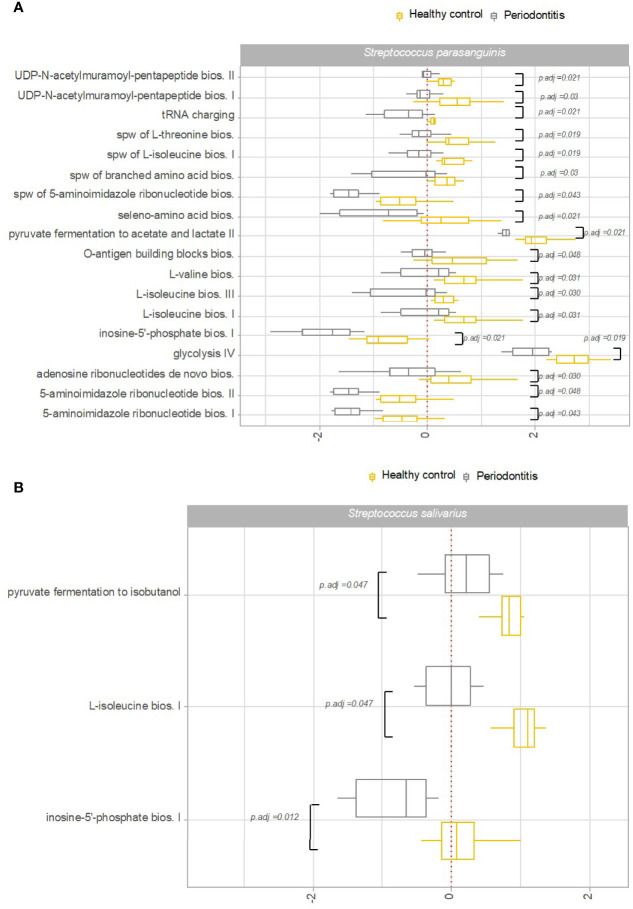
Pathway expression of *Streptococcus* species in saliva in periodontitis versus oral health. Pathways identified with significantly different log2(RNA/DNA) in saliva from patients with periodontitis versus oral health, with each dot representing a specific sample. **(A)** *Streptococcus parasanguinis*, **(B)** *Streptococcus salivarius*. Boxplots display first and third quartiles, median. Whiskers display 1.5 * IQR. Color coding: yellow: healthy control, grey: periodontitis.

**Figure 7:**
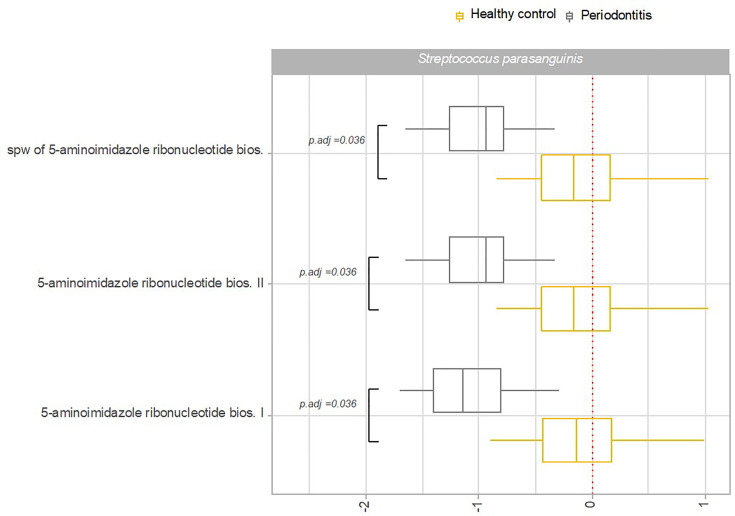
Pathway expression of *Streptococcus* species in tongue biofilm in periodontitis versus oral health. Pathways identified with significantly different log2(RNA/DNA) in tongue biofilm from patients with periodontitis versus oral health with each dot representing a specific sample. *Streptococcus parasanguinis*. Boxplots display first and third quartiles, median. Whiskers display 1.5 * IQR. Color coding: yellow: healthy control, grey: periodontitis.

Transcriptional activity of specific pathways in saliva by *S. parasanguinis* and *S. salivarius* was significantly associated with oral health status. Specifically, transcriptional activity of 18 pathways expressed by *S. parasanguinis* and 3 pathways expressed by *S. salivarius* was significantly less transcribed in saliva from patients with periodontitis versus healthy controls (**Figures 6A, B**). In addition, three pathways expressed by *S. parasanguinis* were identified with significantly less transcriptional activity in tongue biofilm collected from periodontitis patients (**Figure 7**). Finally, no specific pathways were identified with significantly different transcriptional activity in subgingival plaque from periodontitis patients versus healthy controls.

## Discussion

The purpose of the present study was to unravel if transcriptional activity of predominant *Streptococcus* species associates with ecological conditions determined by oral site and periodontitis. The main finding was that transcriptional activity of specific *Streptococcus* species differed significantly at various oral sites and in healthy oral conditions versus periodontitis. To the best of our knowledge, this is the first study to perform paired metagenomics and metatranscriptomics to characterize and compare transcriptional activity of predominant *Streptococcus* species in clinical samples collected from multiple oral compartments in healthy conditions and periodontitis.

The first important finding was that in health, the predominant *Streptococcus* species expressed a site-specific colonization pattern of the tongue and tooth surface. Accordingly, *S. parasanguinis*, *S. salivarius* and *S. infantis* were identified with high relative abundance in tongue biofilm, but almost completely absent in subgingival biofilm in the same individual. In contrast, *S. oralis*, *S. sanguinis* and *S. gordonii* expressed a complete opposite pattern with high abundance in subgingival biofilm in combination with near absence in tongue biofilm (**Figure 1**). Whole genome sequencing of most *Streptococcus* species, including *parasanguinis* and salivarius has been performed, and comparative genomics of genus *Streptococcus* has revealed that the average *Streptococcus* genome is comprised of approx. 1991 genes, out of which the core genome that is shared by all strains is approx. 369 genes (Gao et al., 2014). In addition, phylogenomic analyses of 138 *Streptococcus* genomes revealed branching of *Streptococcus* strains in two distinct groups, with one population comprised by *Streptococcus pyogenes*, *bovis*, *mutans* and *salivarius*, and the other population composed of *Streptococcus mitis*, *anginosus* and unknown groups (Gao et al., 2014). Interestingly, this branching was confirmed in the present study by site-specific preferences of *S. salivarius* versus *S. mitis* and *mutans* (**Figure 1**). Thus, the antagonistic colonization patterns most likely represent a combination of species-specific ecological preferences and different virulence factors, which facilitate interaction with surface adhesion molecules differing between the teeth and the tongue.

Species-specific transcriptional activity was associated with oral site in healthy conditions. In general, higher transcriptional activities were observed in saliva versus tongue biofilm, whereas higher activities were seen in subgingival plaque as compared to saliva (**Figures 2A, B**). In addition, major differences were noted in transcriptional activity of *S. parasanguinis, S. infantis* and *S. salivarius* in saliva versus tongue, but the association of site-specific ecological conditions and transcriptional activity in health differed significantly between these *Streptococcus* species (**Figure 2A**). Accordingly, 11, 4 and 2 pathways expressed by *S. parasanguinis*, *S. infantis* and *S. salivarius*, respectively, were identified with significantly higher transcriptional activity in saliva versus tongue biofilm (**Figures 3A–C**). Besides being identified with the most significant pathways, *S. parasanguinis* was also recorded with a characteristic activity pattern, with 10 out of 11 pathways identified being more active (log2(RNA/DNA)>0) in saliva and less active (log2(RNA/DNA)<0) in tongue biofilm (**Figure 3A**). Notably, most pathways identified as transcriptionally active were involved in either synthesis of peptidoglycan (UDP-N-acetylmuramoyl-pentapeptide biosynthesis), amino acid biosynthesis, or carbohydrate metabolism (glycolysis), which may be involved in supply of energy and molecules for biosynthetic processes and bacterial growth. Importantly, the main difference between saliva and tongue biofilm is oxygen availability and biofilm maturity stage, with the tongue being characterized by a mature biofilm thriving in anaerobic conditions (Segata et al., 2012).

Therefore, data which shows significantly higher transcriptional activity of *S. parasanguinis* of relevant metabolic pathways involved in bacterial growth and carbohydrate degradation, in aerobic versus anaerobic conditions, highlights the important role of the facultative anaerobe *S. parasanguinis* in initial biofilm formation performed in anaerobic environment. Indeed, this is in line with the current view of *S. parasanguinis* as one of the primary colonizers of the dental biofilm (Kolenbrander et al., 2000).

In general, minor differences were observed in species-specific transcriptional activity in saliva versus subgingival plaque in healthy conditions. Accordingly, only 3 pathways expressed by *S. oralis* and 1 pathways expressed by *S. sanguinis* were seen with significantly higher activity in subgingival plaque than in saliva (**Figures 4A, B**), whereas no significant pathways expressed by *S. gordonii* were identified. Importantly, the ecological conditions found in the subgingival environment in healthy conditions are in general more comparable with saliva, with aerobic surroundings on the tooth surface versus anaerobic conditions at the tongue surface (Wake et al., 2016; Hall et al., 2017). Likewise, as long as regular oral hygiene is maintained, the maturity stage of the biofilm found in the subgingival environment is immature as compared to that of the tongue (Belstrøm et al., 2018). Therefore, the finding of more comparable bacterial activity profiles in subgingival plaque and saliva versus saliva and tongue demonstrates the resilience of the permanent microbiota, in healthy conditions, to adapt to the ecological surroundings (Rosier et al., 2018).

An important finding, however, was that the 2 out of 3 pathways identified with different expression by *S. oralis* in subgingival plaque and saliva were involved in carbohydrate metabolism (glycolysis and glycogen biosynthesis). Therefore, data suggest that *S. oralis* is more efficient in carbohydrate degradation and storage, when being part of a polymicrobial biofilm, than in the planktonic environment offered by saliva. Indeed, this finding is of particular interest, since *S. oralis* has been reported to have an important role in homeostasis of oral biofilms (Thurnheer and Belibasakis, 2018). The capacity to synthesize intra- or extracellular polysaccharides or both, can promote survival of the bacteria and enables acid generation during starvation, in the absence of carbohydrate intake. Importantly, excessive carbohydrate metabolism of *Streptococcus* species, which leads to frequent critical decrease in pH of the biofilm, is the central act in the development of dental caries (Pitts et al., 2017). It is therefore interesting that we have recently demonstrated that a coordinated perturbation of short-term sugar stress induces a significant increase in relative abundance of *Streptococcus* species in the oral cavity even though regular oral hygiene is maintained (Lundtorp-Olsen et al., 2021). Indeed, carbohydrate metabolism of *Streptococcus* species may also be beneficial to maintenance of biofilm homeostasis, in conditions with transient increase in pH caused by local inflammation of the tooth supporting tissue – periodontitis (Nyvad and Takahashi, 2020). Thus, these findings underline the need of biofilm control through regular oral hygiene in combination with limited carbohydrate availability to control the doubled edged nature of carbohydrate metabolism performed by the predominant *Streptococcus* species (Philip et al., 2018).

Transcriptional activity in saliva was significantly associated with periodontitis, where significantly lower activities of *S. parasanguinis, S. salivarius, S. infantis, S. gordonii* and *S. oralis* were recorded. Periodontitis was also moderately associated with transcriptional activity of the tongue biofilm, as expressed by significantly lower activity of *S. parasanguinis, S. salivarius* and *S. oralis*. Finally, periodontitis was less associated with transcriptional activity of subgingival plaque, with *S. sanguinis* and *S. oralis* as the only species being significantly associated with oral health status (**Figure 5**). The finding of a significantly lower transcriptional activity of *Streptococcus* species in saliva is in concert with a previous study, where we identified lower bacterial activity of firmicutes in saliva from patients with periodontitis, versus healthy controls (Belstrøm et al., 2017). However, in that previous report we did not have the sequencing depth to reach species-level characterization of transcriptional activity. Thus, this study adds significantly to this finding by identification of transcriptional activity of specific members of genus *Streptococcus* as the part of the salivary microbiota to associate with periodontal status. It is however surprising that periodontitis was only minimally associated with transcriptional activity of *Streptococcus* species in subgingival plaque, when considering that this biofilm is the one found in close proximity to the local inflammation of the periodontal tissues (Kumar et al., 2021). Indeed, several studies have used metagenomics to identify a pathogenic signature of the subgingival microbiota in periodontitis (Wang et al., 2013; Shi et al., 2015). In that respect, one explanation might be that the ecological conditions, such as a decrease in oxygen availability and an increase in pH, which periodontitis exerts locally are more associated with increased activity of proposed periodontal pathogens (Duran-Pinedo et al., 2014), rather than a decrease in activity of *Streptococcus* species.

Another important finding was that periodontitis was differentially associated with transcriptional activity of predominant *Streptococcus* species in saliva. Accordingly, 18 pathways expressed by *S. parasanguinis* and 3 pathways expressed by *S. salivarius* were recorded with significantly less activity in patients with periodontitis, whereas the activity of specific pathways of the remaining predominant *Streptococcus* species was not associated with periodontitis (**Figures 6A, B**). Interestingly, transcriptional activity of both *S. parasanguinis* and *S. salivarius* of the same 3 pathways involved in 5-aminoimidazole ribonucleotide biosynthesis were associated with periodontitis (**Figures 6A, B**). In addition, *S. parasanguinis* was the only *Streptococcus* species identified in tongue biofilm, with activity that was associated with periodontitis (**Figure 7**). Notably, the majority of pathways expressed by *S. parasanguinis*, which were recorded with less activity in saliva from patients with periodontitis, were also involved in peptidoglycan and amino acid biosynthesis, and 10 out of 11 pathways that were identified with higher transcriptional activity in saliva versus tongue biofilm in health, were also associated with periodontitis. In addition, a significantly lower transcriptional activity of glycolysis expressed by *S. parasanguinis* was noted in saliva from patients with periodontitis versus healthy controls (**Figure 6A**).

Interestingly, data therefore suggest that transcriptional activity of *S. parasanguinis*, in contrast to the remaining predominant *Streptococcus* species characterized in the present study, is heavily associated with ecological characteristics offered by both oral site as and presence of chronic oral inflammation. Indeed, *S. parasanguinis* in saliva from orally healthy individuals were identified with significantly higher transcriptional activity of pathways involved in peptidoglycan and amino acid biosynthesis as well as glycolysis, than *S. parasanguinis* in concomitant tongue samples. On the contrary, conditions of periodontitis was associated with significantly less expression of the same pathways by *S. parasanguinis* in saliva. When considering that *S. parasanguinis* was highly abundant in both saliva and tongue biofilm (**Figure 1**), changes in transcriptional activity of this particular species would most likely affect the phenotypic profile of the total biofilm community with potential to affect oral homeostasis. Importantly, the two major oral diseases, i.e. dental caries and periodontitis are multifactorial diseases, where compositional changes of the oral microbiota play a critical role in pathogenesis. However, the specific species involved in the two diseases are critically different and possess almost antagonistic virulence factors (Marsh and Zaura, 2017). It is therefore noteworthy that *S. parasanguinis* is one of the only oral bacterial species that has been reported to associate with both caries (Aas et al., 2008), and has been given a role in periodontitis as accessory pathogen to the periodontal pathogen *Aggregatibacter actinomycemcomitans (* Fine et al., 2013). Thus, data from the present study illuminates the versatility of *S. parasanguinis* to thrive in areas with critically different ecological conditions, which could be an explanation as to why, this particular *Streptococcus* species associates with both dental caries and periodontitis.

The present study has several limitations, including the relative low sample size. However, the sample size was comparable with other metagenomics studies on the oral microbiota (Kumar et al., 2021). Studies with much larger sample sizes have been performed on the oral microbiota using more cost effective molecular methods such as microarray and 16S sequencing (Willis and Gabaldón, 2020), but these methods do not provide any information on bacterial activity, which was the purpose to address in the present study. Indeed, the sequencing depth of the current dataset in combination with concomitant sampling was sufficient to discriminate transcriptional activity of selected *Streptococcus* species. However, this was only possible as the selected species were predominant and identified with relative abundance ≥ 2% in all samples (**Figure 1**). Accordingly, a larger number of samples or an increased sequencing depth would be needed, to obtain species-level resolution of transcriptional activity of more peripheral members of the oral microbiota, including proposed periodontal pathogens such as *Porphyromonas gingivalis*, which is usually identified with relative abundance ≤ 1% (Damgaard et al., 2019). Also, current smokers were included in both the healthy and the periodontitis group. However, a comparable number of current smokers were present in the two groups studied. Smoking has been demonstrated to associate with characteristics of the subgingival microbiota in both healthy individuals and patients with periodontitis (Bizzarro et al., 2013; Mason et al., 2015), whereas the effect on the salivary microbiota is probably minimal (Belstrøm et al., 2014). Importantly, smoking has not been shown to associate with relative abundance or activity of predominant *Streptococcus* species, which is why smokers were not excluded in the present study. The main strength of the present study was concomitant sampling from multiple oral sites from both healthy individuals and patients with periodontitis, which facilitated the possibility to study the association of both site-specific ecological conditions and oral inflammation with transcriptional activity of the predominant members of the oral microbiota – the oral streptococci.

In conclusion, data from the present study clearly demonstrates the association of site-specific ecological conditions and presence of chronic oral inflammation with transcriptional activity of the predominant *Streptococcus* species of the oral microbiota. In particular, pathways expressed by *S. parasanguinis* being involved in peptidoglycan and amino acid biosynthesis and glycolysis were identified to be significantly associated with oral site and inflammation status. Further studies are needed to reveal if different activity patterns of *S. parasanguinis* link this particular bacterial species with dental caries and periodontitis.

## Data Availability Statement

The datasets presented in this study can be found in online repositories. The names of the repository/repositories and accession number(s) can be found below: https://www.ncbi.nlm.nih.gov/, PRJNA678453.

## Ethics Statement

The study was approved by the regional ethical committee (H-16016368) and reported to the local data authorization of University of Copenhagen (SUND-2018-8). The patients/participants provided their written informed consent to participate in this study.

## Author Contributions 

DB, FC, and MG defined the bioinformatic and biostatistical analyses, which were performed by FC. DB, FG, and MG wrote the first draft of the paper, which was critically revised by MM, MS, and CS. All authors contributed to the article and approved the submitted version.

## Funding

Financial support was provided solely by SCELSE.

## Conflict of Interest

The authors declare that the research was conducted in the absence of any commercial or financial relationships that could be construed as a potential conflict of interest.

## Publisher’s Note

All claims expressed in this article are solely those of the authors and do not necessarily represent those of their affiliated organizations, or those of the publisher, the editors and the reviewers. Any product that may be evaluated in this article, or claim that may be made by its manufacturer, is not guaranteed or endorsed by the publisher.
